# Synthesis and *in vivo* evaluation of PEG-BP–BaYbF_5_ nanoparticles for computed tomography imaging and their toxicity†

**DOI:** 10.1039/d0tb00969e

**Published:** 2020-07-11

**Authors:** Cinzia Imberti, Thais Fedatto Abelha, Yong Yan, Jaclyn Lange, Xianjin Cui, Istvan Szanda, Vicky Goh, Lea Ann Dailey, Rafael T. M. de Rosales

**Affiliations:** Department of Imaging Chemistry & Biology, School of Biomedical Engineering & Imaging Sciences, King's College London, St Thomas’ Hospital London SE1 7EH UK rafael.torres@kcl.ac.uk; School of Pharmacy, University of Nottingham, University Park Nottingham NG7 2RD UK; School of Chemistry, University of Nottingham, University Park Nottingham NG7 2RD UK; Department of Cancer Imaging, School of Biomedical Engineering & Imaging Sciences, King's College London, St Thomas’ Hospital SE1 7EH London UK; Department of Pharmaceutical Technology and Biopharmacy, University of Vienna, Althanstraße 14 1090 Vienna Austria; London Centre for Nanotechnology, King's College London, Strand Campus London WC2R 2LS UK

## Abstract

Computed tomography (CT) is one of the most widespread imaging techniques in clinical use worldwide. CT contrast agents are administered to improve soft tissue contrast and highlight blood vessels. However, the range of CT contrast agents available in the clinic is limited and they suffer from short-circulation times and low k-edge values that result in the need for high doses for *in vivo* applications. Nanomaterials containing a mixture of electron-dense elements, such as BaYbF_5_ nanoparticles, have shown promise as more efficient CT contrast agents, but they require biocompatible coatings for biomedical applications. Here, we explore the use of a bifunctional PEG polymer (5 kDa) containing a terminal bisphosphonate (BP) anchor for efficient binding to the surface of BaYbF_5_ nanomaterials. The resulting PEG(5)-BP–BaYbF_5_ nanoparticles were synthesized and characterized using TEM, DLS, TGA, XRD and *Z*-potential measurements. Their *in vitro* stability was verified and their ability to produce CT contrast in a wide range of X-ray energies, covering preclinical and clinical scanners, was demonstrated. *In vitro* toxicity studies with PEG(5)-BP–BaYbF_5_ in the phagocytic pro-monocytic human cell line U937 did not identify toxic effects, even at high concentrations (30 mM). *In vivo*, PEG(5)-BP–BaYbF_5_ exhibited efficient CT contrast for angiography imaging, highlighting blood vessels and vascular organs, and long circulation times as expected from the PEG coating. However, at late time points (48 h), *in vivo* toxicity was observed. While the causes could not be completely elucidated, *in vitro* studies suggest that decomposition and release of Yb^3+^ and/or Ba^2+^ metal ions after decomposition of PEG(5)-BP–BaYbF_5_ may play a role. Overall, despite the promising CT contrast properties, our results suggest that BaYbF_5_ nanomaterials may suffer from significant long-term toxicities.

## Abstract 2

PEG(5)-BP–BaYbF_5_ nanoparticles provide superior CT contrast and circulation time compared to clinically-used iodinated molecules, but suffer from unexpected *in vivo* toxicity.

## Graphical abstract

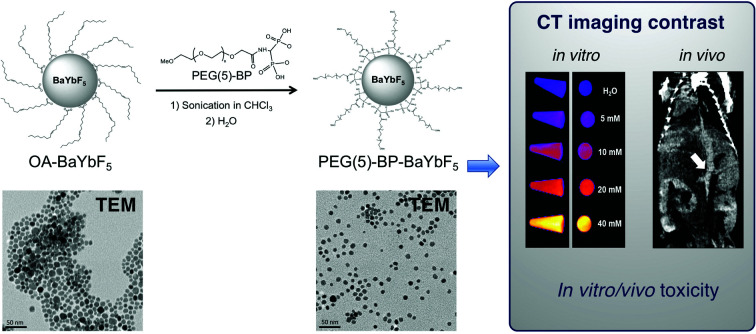

## Introduction

Computed tomography (CT) is one of the most widespread imaging techniques in clinical use worldwide.^1^ It is based on the differential attenuation of X-ray beams by different tissues according to their attenuation coefficient *κ*. For a particular material, *κ* depends on its elemental composition, being larger for high atomic number (*Z*) elements (*κ* ∝ *Z*^3^), and on the energy of the incident X-ray beam. The attenuation coefficient of a material sharply increases at a specific energy, referred to as the k-edge.^2^ At the operating voltage employed in clinical CT instruments (80–150 kVp, depending on the patient),^3^ bones, air cavities and soft tissues can be visualized and distinguished, but only minor differentiation between different soft tissues is achieved.

The low soft-tissue contrast properties of CT can be circumvented with the use of contrast agents. These should be rich in chemical elements with a high *Z* and large attenuation coefficient values to enhance the contrast between different tissues.^1^ Two types of contrast agents are currently available for clinical use: the oral BaSO_4_ suspension (*Z*_Ba_ = 56), limited to luminal gastrointestinal imaging, and parenterally administered, iodinated aromatic molecules (*Z*_I_ = 53), representing the gold standard in clinical CT contrast agents.^4,5^ Despite their widespread use, iodinated contrast agents present some drawbacks, such as short circulation times (from seconds to minutes post-injection), hindering their application in angiography and targeted imaging.^6^ Additionally, their k-edge value (33 keV) is significantly lower than the X-ray energies employed in CT (50–70 keV for a standard X-ray tungsten tube operating at 150 kVp). Hence, larger doses of contrast agents are required to achieve acceptable contrast enhancement, potentially leading to serious adverse effects such as deterioration of renal function.^7,8^

The use of nanoparticles (NPs) as CT contrast agents represents a powerful strategy to address these shortcomings.^9–14^ NPs are capable of packing together many atoms (thousands) with a high *Z* within a small volume, achieving a high contrast/carrier ratio. When combined with a stealth coating, their size (typically between 10 and 500 nm) promotes longer circulation times required for high-spatial resolution angiography and cancer detection. Notably, long-circulating nanoparticles are known to accumulate in some tumors based on the enhanced permeability and retention effect (EPR).^15^ In addition, nanoparticles can be functionalized, thus becoming a suitable platform to design molecular imaging probes targeting specific biomarkers.^16,17^*In vivo* application of many NP systems, however, suffers from some drawbacks, including poor colloidal stability in physiological media, and biological barriers such as uptake from the mononuclear phagocytic system (MPS) through opsonisation.^3,18^ Coating NPs with suitable hydrophilic biocompatible polymers, such as polyethylene glycol (PEG), is an important technique to overcome these issues. Such a coating improves colloidal stability owing to the reduced interaction amongst NPs (responsible for aggregation) and *in vivo* opsonization processes (responsible for MPS uptake), resulting in long-circulating and low-toxicity NPs.^19^

Several NP platforms have been explored as CT contrast agents.^9,11–14^ Iodinated nanoparticles have been developed to increase the circulation time of iodine small-molecules,^20^ but the low k-edge energy of iodine still remains a limitation. More recently, research efforts have focused on different types of nanoparticulate contrast agents such as metal-based nanoparticles. Gold (Au) NPs have shown longer circulation times *in vivo* and X-ray attenuation comparable to iodinated agents,^9,21^ but their clinical applicability may be limited by the price of this element. Bismuth (Bi) is less expensive and displays a high attenuation coefficient and low toxicity. Bi_2_S_3_ nanoparticles coated with a biocompatible poly(vinylpyrrolidone) polymer demonstrated high circulation times and contrast efficacy.^16^ Ytterbium (Yb) has gained attention as a material for nanoparticle-based CT contrast agents.^22–25^ With a k-edge of 61 keV that is within the clinical CT X-ray energy range (50–70 keV), Yb provides a higher intrinsic contrast compared to gold (k-edge: 81 keV) or bismuth (k-edge: 91 keV).^3^ Yb-based nanoparticles have also been investigated in the emergent field of spectral CT, where different elements can be identified by their k-edge energy, and visualized as colored voxels.^3,26^

Binary CT-contrast agents, combining elements with different k-edge values, have been recently developed to address the need of high attenuation capability at different operating CT voltages. These include BaYbF_5_ nanomaterials, in which Ba and Yb provide X-ray attenuation at low and high voltage, respectively. These NPs have been encapsulated in a SiO_2_ core and functionalized with PEG-silane, showing increased contrast and circulation times compared to the clinical standards.^27^ In a different study, oleic acid (OA)–BaYbF_5_:2% Er^3+^ upconversion nanocubes were developed to display near infrared luminescence given by erbium doping. These nanoparticles were coated with the phospholipid conjugate DSPE-PEG2000 to endow them with water dispersibility and they were conjugated to RGD (arginine–glycine–aspartate) peptides to improved targeting of cancer cells.^28^ More recently, core–shell BaYbF_5_:Tm@BaGdF_5_:Yb,Tm nanocrystals, also using DSPE-PEG2000, were investigated as a trimodal contrast agent for upconversion luminescence and magnetic resonance as well as CT.^29^ Polyacrylic acid (PAA) has also been investigated as a coating for BaYbF_5_, whereby Liu *et al.* synthesized PAA–BaYbF_5_:Tm upconversion nanoparticles for multimodal imaging (CT/upconversion luminescence) of the gastrointestinal tract, achieving good contrast capability.^30^ This contrast agent was completely excreted in 2 days after oral administration and exhibited low long-term toxicity, based on the monitoring of animal body weight and behavior as well as histological examination of tissues.^30^ Finally, ligand-free BaYbF_5_:Gd/Er and BaYbF_5_:Tm upconversion nanoparticles have been prepared.^31,32^ Unsurprisingly, *in vivo* evaluation of intravenously (*i.v.*) administered ligand free BaYbF_5_:Tm as a trimodal contrast agent (CT/upconversion luminescence/MRI) revealed a high signal in the liver and spleen likely due to the lack of coating, resulting in the prompt uptake of the NPs by the MPS.^32^

In our search for novel NP coatings for imaging applications, our group developed a novel biocompatible polymer, PEG(5)-BP, composed of a PEG moiety (MeO-PEG, 5 kDa) conjugated to tetra-ethyl aminomethyl-bisphosphonate (BP).^33^ This polymer was successfully used to coat superparamagnetic iron oxide NPs through a ligand-exchange reaction. The BP moiety was able to covalently bind the iron oxide core, generating NPs with high *in vitro* and *in vivo* stability and promising properties as a magnetic resonance imaging (MRI) contrast agent.^33^ We hypothesized that the use of this polymer could be extended to other NPs containing metals with pronounced coordination chemistry, especially lanthanide ions, whose great affinity for phosphate ligands has been shown in the literature.^34–36^ In this work, we investigated the use of PEG(5)-BP polymers as coatings for BaYbF_5_ nanoparticles, leading to the formation of the nanoparticulate contrast agent PEG(5)-BP–BaYbF_5_. The aim of this new nanomaterial was to combine the promising CT contrast enhancing properties of BaYbF_5_ with the high water solubility, *in vivo* stability and *in vivo* stealth properties of the PEG(5)-BP coating. The CT contrast enhancement capability of PEG(5)-BP–BaYbF_5_ was evaluated *in vitro* and *in vivo*, revealing a long blood circulation time but also, unexpectedly, *in vivo* toxicity, not previously reported for BaYbF_5_ nanoparticles.

## Results and discussion

### Synthesis and characterization of PEG(5)-BP–BaYBF_5_ nanoparticles

**Fig. 1:**
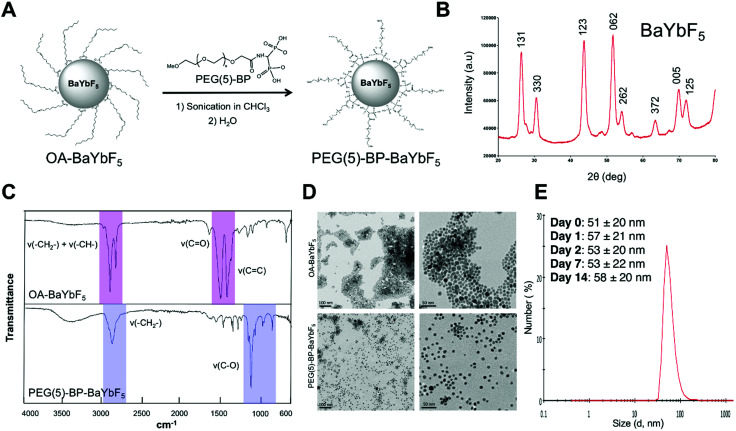
(A) Schematic representation of the synthesis of PEG(5)-BP–BaYbF_5_ from OA–BaYbF_5_. (B) Powder X-ray diffraction pattern of OA–BaYbF_5_, the peaks are labelled according to the corresponding lattice planes (obtained by comparison with the JCPDS database – 46-0039). (C) IR spectra of OA–BaYbF_5_ and PEG(5)-BP–BaYbF_5_ nanoparticles confirming the successful displacement reaction. The signals attributable to the OA-capping are highlighted in pink, while the PEG-related signals are colored in violet. (D) TEM images of OA–BaYbF_5_ (top row, dispersion in chloroform) and PEG(5)-BP–BaYbF_5_ (bottom row, dispersion in water) at different scales (100 nm, left; 50 nm, right), showing the inorganic core of the NPs, unaffected by the different coatings. High resolution versions of these images have been included in the ESI† Fig. S1. (E) Dynamic light scattering size distribution of PEG-BP–BaYbF_5_ nanoparticles and mean values for the hydrodynamic diameter (*D*_H_, average of three measurements) over two weeks (in water/phosphate buffered saline and room temperature).

PEG(5)-BP–BaYBF_5_ was synthesized following our previously reported ligand-displacement method (Fig. 1A).^33^ First, oleic acid capped nanoparticles (OA–BaYbF_5_) were synthesized exploiting a modified literature method,^27^ and characterized by powder-XRD, matching the reported data and pattern for cubic BaYF_5_ crystals (JCPDS No. 46-0039) (Fig. 1B). Subsequently, PEG-bisphosphonate (PEG(5)-BP) was introduced through a displacement reaction (Fig. 1A) where PEG(5)-BP (80 mg) was added to a dispersion of OA–BaYbF_5_ in chloroform (40 mg in 1 mL) and the open reaction vial was sonicated until complete solvent evaporation. Excess PEG(5)-BP was used during the reaction in order to maximize the yield of the displacement reaction and the density of the coating on the NP surface. Unreacted PEG(5)-BP was removed by size-exclusion filtration. Notably, compared to other PEGylation methods, this method is fast and does not require heating.^33^ The success of this reaction was evidenced by a change in the dispersibility of the nanoparticles, now easily dispersed in water due to the hydrophilic PEG chains on the surface of the BaYbF_5_ NPs. OA displacement by PEG was confirmed by IR spectroscopy data (Fig. 1C), with the spectrum of OA–BaYbF_5_ exhibiting all the expected vibrations of oleic acid, which were absent in the IR spectrum of PEG(5)-BP–BaYbF_5_. Conversely, the IR spectrum of the latter displayed all the expected vibrations for PEG (*ν*(–CH_2_–) = 2884 cm^−1^; *ν*(C–O) + *ρ*(–CH_2_–) = 1113 cm^−1^; *ρ*(–CH_2_–) + *τ*(–CH_2_–) = 964 cm^−1^; *τ*(–CH_2_–) + *ν*(C–O) = 843 cm^−1^). Notably, in contrast to the previously reported synthesis of PEGylated BaYbF_5_,^27,28^ where the use of intermediate layers was necessary to anchor PEG onto the surface of the NPs, here, the BP moiety directly binds to the surface of the metallic nanoparticles *via* covalent coordination bonds. To the best of our knowledge, this is the first time that bisphosphonates (BPs) have been used as anchors for coating lanthanide-based nanoparticulate materials.

The diameter of the inorganic core for both NPs was measured using TEM based on the analysis of 200 NPs (Fig. 1D and Fig. S1, ESI†). TEM imaging of the PEG(5)-BP–BaYbF_5_ NPs revealed a 9.5 ± 1.7 nm diameter (mean ± SD), virtually identical to the diameter measured for OA–BaYbF_5_ using the same method (9.3 ± 2.1 nm). This result shows that the dimensions of the NP inorganic core were not affected by ligand displacement and suggests that the PEG(5)-BP ligand does not etch the NP surface.

Colloidal stability in relevant media is an essential parameter for the biomedical applications of NPs. PEG(5)-BP–BaYbF_5_ NPs were found to be colloidally stable in water and phosphate buffered saline (PBS) for over two weeks, as confirmed by dynamic light scattering (DLS) measurements, highlighting the efficacy of PEG(5)-BP for sterically stabilizing NPs in aqueous media. In particular, DLS measurements revealed an average hydrodynamic diameter (*D*_H_) of 51 ± 20 nm (mean ± SD) for the NPs in water over 14 days (Fig. 1E). The higher hydrodynamic diameter measured by DLS – compared to the TEM value for the inorganic core – accounts for the PEG-coating, and the large standard deviation of its distribution could be attributed to the different conformations that PEG chains can take in solution. Notably, the *D*_H_ measured for PEG(5)-BP–BaYbF_5_ does not match a fully extended conformation of the PEG polymers, which would result in a *D*_H_ of *ca.* 85 nm (considering the theoretical 38 nm length of an extended PEG(5)-BP chain). This suggests that the PEG layer in PEG(5)-BP–BaYbF_5_ NPs is present in an expanded coil conformation, with the PEG chains folded to half their extended length. A similar effect has been found when PEG(5)-BP was used to coat iron oxide-based nanoparticles such as USPIOs,^33^ suggesting that this is a feature of the PEG(5)-BP coating. The PEG density on the surface of PEG(5)-BP–BaYbF_5_ was calculated from thermogravimetric analysis (TGA) measurements (Fig. S2, ESI†), which revealed that 37% of the total mass of PEG(5)-BP–BaYbF_5_ was PEG(5)-BP. This corresponds to an average of 196 PEG(5)-BP per nanoparticle or 0.7 PEG per nm^2^, considering BaYbF_5_ nanoparticles as perfect 9.5 nm spheres with a density intermediate between that of BaF_2_ and YbF_3_. This result implies that PEG-BP is present in the brush regime, taking into account the fact that the maximum theoretical number of PEG per NP is 333 (see the ESI† for calculations). A *Z*-potential of −2.1 ± 0.4 mV (average of 3 measurements ± SD) was measured in 10% PBS (pH = 7.4). The almost neutral *Z*-potential found at physiological pH is typical of PEG-coated nanoparticles and should allow them to evade the *in vivo* opsonization process and following MPS sequestration, thus prolonging their blood half-life.^37^ Attempts to evaluate this aspect *in vitro* by measuring the *D*_H_ in the presence of serum proteins were unsuccessful. It should be noted, however, that despite its stealth coating, it is likely that PEG(5)-BP–BaYbF_5_ will attract serum proteins and form a protein corona *in vivo* that will contribute to its pharmacokinetics and biodistribution, as demonstrated for other nanomaterials.^38^

### *In vitro* CT imaging with PEG(5)-BP–BaYbF_5_

**Fig. 2:**
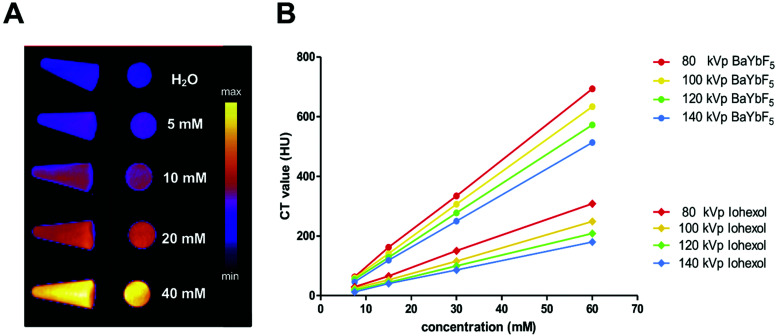
(A) Phantom-CT images of PEG(5)-BP–BaYbF_5_ dispersed in water (0–40 mM), obtained with the preclinical scanner (55 kVp) presented in color scale. (B) Phantom–CT values, obtained using the clinical CT scanner, comparing CT-contrast efficiency of Iohexol and PEG(5)-BP–BaYbF_5_ (dispersed in water) over a range of concentrations (0–60 mM) and CT-operating potential (80–140 kVp).

To verify the contrast enhancement capability, a phantom CT imaging study was performed using both preclinical and clinical CT scanners (Fig. 2). Vials with increasing concentrations of PEG(5)-BP–BaYbF_5_ were imaged in a preclinical CT scanner (55 kVp), demonstrating their contrast enhancement at this operating voltage (Fig. 2A). Clinical CT scanners, however, operate at different ranges of X-ray voltages; hence, we also evaluated the contrast capabilities of PEG(5)-BP–BaYbF_5_ nanoparticles in a clinical CT scanner (Fig. 2B), in comparison with Iohexol, which is an iodinated CT contrast agent currently in clinical use and with proven angiography contrast properties in both humans and rodents.^39–41^ For both contrast agents, CT scans were performed in a range of operating voltages (80–140 kVp) to take into account the fact that this parameter is dependent on patient size. As shown in Fig. 2, CT values for both PEG(5)-BP–BaYbF_5_ and Iohexol are linearly dependent on the concentration of the contrast agent and decrease upon increasing the operating voltage. Notably, PEG(5)-BP–BaYbF_5_ NPs possess a higher contrast enhancement capability than Iohexol, at any concentration and operating voltage, reaching similar values to those reported by Liu *et al.* for BaYbF_5_@SiO_2_@PEG.^27^

### Evaluating the *in vitro* cytotoxicity of PEG(5)-BP–BaYbF_5_

**Fig. 3:**
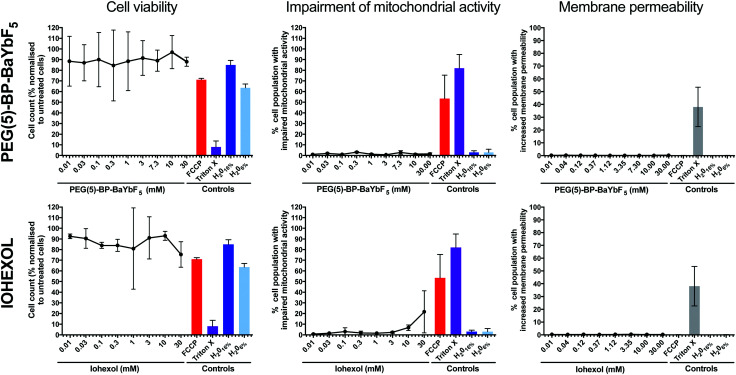
Cytotoxicity data for PEG(5)-BP–BaYbF_5_ nanoparticles (top row) and Iohexol (bottom row) in U397 cells, including cell viability for the tested compounds, percentage of cells with impaired mitochondrial activity and membrane permeability. Each data point represents the average of 3 independent measurements ± SD. For each sample, the mitochondrial toxin FCCP and the membrane permeabilizer Triton X were used as positive controls, and water was used as a negative control.

PEG(5)-BP–BaYbF_5_ NPs were tested for cytotoxicity, in comparison with the clinical CT contrast agent Iohexol, using the human cell line U937.^42^ The human pro-monocytic cell line U937 is a clinically relevant model for differentiation into macrophages and cytotoxic assessment. Biocompatibility assays in phagocytic cells, such as macrophages, are relevant because nanoparticles are known to mainly interact with these cell types following intravenous injection.^43–45^ U937 cells exhibit many characteristics of monocytes,^46,47^ and incubation with phorbol 12-myristate 13-acetate (PMA) triggers differentiation to obtain a macrophage-like phenotype.^48^ Measurements of cell viability, as a general indicator of cellular death, were performed, as well as those of impaired mitochondrial activity and increased membrane permeability, to account for apoptosis and necrosis, respectively (Fig. 3). The mitochondrial toxin, carbonyl cyanide 4-(trifluoromethoxy)phenylhydrazone (FCCP), and the membrane permeabilizer, Triton X, were used as positive controls, while water was used as a negative control. Both PEG(5)-BP–BaYbF_5_ nanoparticles and Iohexol were found to be non-cytotoxic and had no effect on cell membrane permeability over the entire concentration range tested (0.01–30 mM), supporting their use for *in vivo* CT imaging applications. In addition, PEG(5)-BP–BaYbF_5_ showed no impairment of mitochondrial activity at concentrations as high as 30 mM, while Iohexol showed increasing levels of mitochondrial impairment at concentrations >3 mM. This result could be due to a higher cell membrane permeability of Iohexol, a relatively small neutral molecule (*M*_w_ = 821 g mol^−1^), compared to the large and hydrophilic PEG(5)-BP–BaYbF_5_ nanoparticles. Further experiments that are beyond the scope of this study would be required to test this hypothesis.

### *In vivo* evaluation of PEG(5)-BP–BaYBF_5_ as an angiography CT contrast agent

**Fig. 4:**
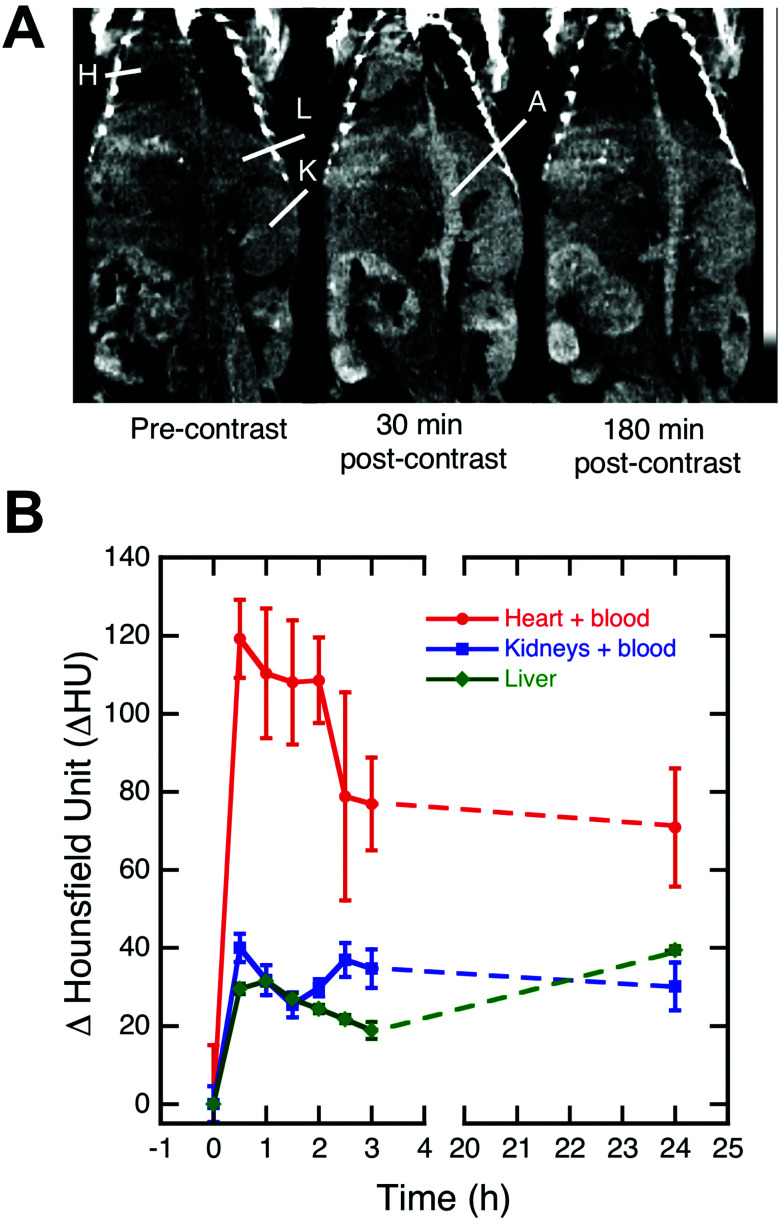
(A) Coronal CT images centered at the descending aorta of a healthy mouse before and after intravenous injection (in saline) of PEG(5)-BP–BaYbF_5_. Clear CT contrast is visible after injection in the heart (H), descending aorta (A), kidneys (K) and liver (L). (B) Image-based analysis (*n* = 2) of the CT contrast (ΔHU) over time in several organs from *t* = 0 (pre-injection) to *t* = 24 h post-injection. Error bars represent the standard deviation from two independent image-based analyses.

Encouraged by the physicochemical properties, lack of cell toxicity *in vitro*, high X-ray attenuation capability, and colloidal stability of PEG(5)-BP–BaYbF_5_, an *in vivo* imaging experiment was performed to evaluate its potential as a CT contrast agent for angiography. Fig. 4A shows coronal view CT images, centered at the descending aorta, of a healthy mouse at different time points after injection of saline dispersed PEG(5)-BP–BaYbF_5_ (133 mM, 100 μL). Notably the CT contrast (measured in Hounsfield units) achieved for the heart, blood vessels and vascular organs such as the kidneys and liver increased after injection, indicating the presence of circulating PEG(5)-BP–BaYbF_5_ nanoparticles. Remarkably, the contrast in the heart/blood and kidneys, after peaking within 2 h post-injection, was retained for at least 24 h, indicating long blood circulation times as expected from the physicochemical properties of PEG(5)-BP–BaYbF_5_ (*vide supra*). A similar extended circulation time effect was also identified when coating iron oxide nanoparticles with PEG(5)-BP.^33^ An increase in CT signal from 3 to 24 h in the liver was expected as this is the most common excretion organ for nanoparticulates. In our attempt to image longitudinally to evaluate the long term *in vivo* biodistribution of PEG(5)-BP–BaYbF_5_, the animals were unexpectedly found dead at 48 h post-injection. Accordingly, further *in vivo* evaluation of the NPs was immediately interrupted.

### Evaluating the *in vitro* cytotoxicity of Ba^2+^, Yb^3+^ and PEG(5)-BP

**Fig. 5:**
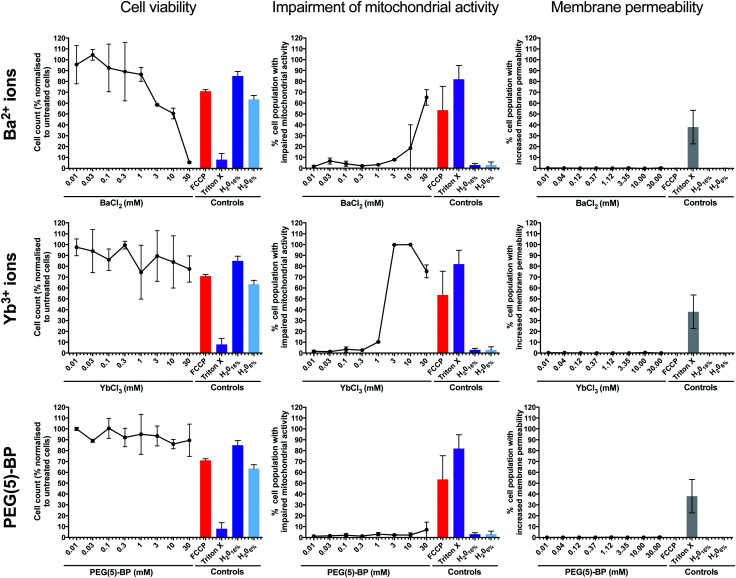
*In vitro* cytotoxicity data for Ba^2+^ ions (top row), Yb^3+^ ions (middle row) and PEG(5)-BP (bottom row) in U397 cells, including cell viability for the tested compounds, percentage of cells with impaired mitochondrial activity and membrane permeability. Each data point represents the average of 3 independent measurements ± SD. For each sample, the mitochondrial toxin FCCP and the membrane permeabilizer Triton X were used as positive controls, and water was used as a negative control and to account for the dilution of cell medium with the sample vehicle.

We hypothesized that the most likely cause of PEG(5)-BP–BaYBF_5_ toxicity was its gradual accumulation in macrophages of the organs of the mononuclear phagocytic system (*e.g.* liver) *in vivo* – as commonly observed for nanoparticles of this size – degradation, and release of its components. Therefore, the same *in vitro* toxicity assays in phagocytic U937 cells as described earlier for PEG(5)-BP–BaYBF_5_ were performed for each of its components: PEG(5)-BP and Ba^2+^ and Yb^3+^ ions (Fig. 5). Even at high concentrations (30 mM), no increase in membrane permeability was measured for any of the tested substances, ruling out necrosis as a mechanism for cytotoxicity. However, high concentrations (>1 mM) of Ba^2+^ and Yb^3+^ ions resulted in cytotoxicity, mainly attributable to apoptotic mechanisms, as revealed by a rise in the percentage of cells displaying impaired mitochondrial activity when treated with either metal ion. While the *in vivo* toxicity of different bisphosphonate molecules was previously reported,^49^ our *in vitro* studies show that PEG(5)-BP was non-cytotoxic at all the concentrations tested, suggesting that PEG(5)-BP is not responsible for the toxicity observed.

Taken together, the *in vitro* results suggest that the source of toxicity found *in vivo* with PEG(5)-BP–BaYBF_5_ is the gradual decomposition of the NPs and release of Ba^2+^ and/or Yb^3+^ ions, which, at the high concentrations required to achieve contrast in CT, result in cell toxicity at late time points of the *in vivo* experiment. Importantly, none of the previous studies using BaYBF_5_ systems have reported toxicity effects *in vitro*/*vivo*, despite using similar concentrations as in this study.^27–30,32^ This suggests that the different coating strategy used in this work could be responsible for the observed toxicity as the absence of an intermediate layer between the PEG coating and the NP core may have promoted solvent penetration resulting in leakage of the core metal ions *in vivo*. It should be noted that none of these studies evaluated the *in vitro* toxicity of each single NP component, limiting their assays to the NPs. However, it is also important to note that of all previously reported studies, only one, in which BaYbF_5_ was administered orally, monitored the animals for more than 24 hours after administration.^30^ It is unclear whether the lack of toxicity observed by this last study for up to one month after administration can be attributed to the different pharmacokinetic properties (owing to the different coating strategies compared to our system) of PAA–BaYbF_5_:Tm or rather to the different administration route (oral *vs.* intravenous) used.

## Conclusions

A new nanoparticulate CT contrast agent, PEG(5)-BP–BaYbF_5_, has been developed, which combines the contrast enhancing capability of BaYbF5 nanoparticles^27–32^ with the water solubility and biocompatibility provided by a dense PEG(5)-BP coating.^33^ Differing from previous reports of PEGylated BaYBF_5_, in PEG(5)-BP–BaYbF_5_, the polymer is directly bound to the NP surface thanks to the strong BP–BaYBF_5_ bond, without the need for intermediate layers. TEM characterization revealed a diameter of 9.5 ± 1.7 nm for the inorganic core of PEG(5)-BP–BaYbF_5_ NPs, which is not significantly different from the 9.3 ± 2.1 nm diameter measured for the OA–BaYbF_5_ precursor, and suggests that PEG(5)-BP does not etch the NP surface. A hydrodynamic diameter of 51 ± 20 nm was measured by DLS, allowing us to hypothesize an expanded coil conformation for the PEG chain on the NP surface. TGA studies revealed a dense concentration of PEG(5)-BP on the surface of BaYbF_5_ nanoparticles that results in an almost neutral surface charge, as identified by *Z*-potential measurements.

PEG(5)-BP–BaYBF_5_ demonstrated high stability in PBS over time and efficient CT contrast at different X-ray energies, allowing its use with current preclinical and clinical scanners. PEG(5)-BP–BaYBF_5_ also benefits from stealth properties provided by the stable and dense PEG(5)-BP layer, which resulted in a long *in vivo* circulation time and efficient CT contrast for angiography. This is in contrast to the currently used angiography CT contrast agent Iohexol that suffers from short circulation times. The long circulation times of PEG(5)-BP–BaYbF_5_ should allow for higher spatial resolution CT images with a single dose of contrast.

Despite all the above advantages of PEG(5)-BP–BaYbF_5_, and despite *in vitro* studies failing to identify any toxicity, this nanomaterial proved to be toxic *in vivo* at late time points (*ca.* 48 h). Notably, no toxicity was observed from the moment of the injection until approximately 48 h. Although the reasons for this toxicity could not be completely elucidated, our studies suggest that the late release of Ba^2+^ and/or Yb^3+^ ions, possibly mediated by the PEG(5)-BP coating, after NP uptake in the organs of the MPS (most likely liver) is a probable cause. A potential strategy to avoid early leakage of these heavy metal ions could involve the introduction of an intermediate inorganic layer of a biocompatible material such as silica.^27^ These results highlight the importance of a thorough *in vitro* investigation of the individual components of nanomaterials in relevant phagocytic cells, as well as long term *in vivo* toxicity monitoring of NP-based contrast agents. This is particularly important for contrast agents developed for insensitive imaging modalities such as CT and MRI that require high amounts of material to achieve contrast. Based on this work, we recommend that PEG(5)-BP–BaYbF_5_ should be avoided in further biomedical *in vivo* CT studies. Furthermore, future studies with BaYbF_5_ materials should carefully perform in depth *in vitro* toxicity studies, including apoptosis/necrosis assays with different coatings and core–shell BaYbF_5_ nanoparticle architectures to interrogate the source(s) of toxicity, as well as long-term (>24 h) *in vivo* toxicities.

## Experimental section

### Materials and instrumentation

All the chemicals were obtained from Sigma Aldrich (St Louis, MO, USA) and used without further purification, unless otherwise specified. Iohexol (Omnipaque™) was obtained from GE Healthcare (UK). MitoTracker® Red CMXRos, Image-iT®-DEAD Green™ viability, HCS Cell Mask™ deep red and Hoechst 33342 (trihydrochloride, trihydrate) were purchased from Life Technologies (Oregon, USA). Sucrose was acquired from VWR International Ltd (England, UK) and paraformaldehyde solution 37–41% was acquired from Fisher Scientific (England, UK). A spectrum 100 spectrometer (PerkinElmer) was used to perform IR measurements. Dynamic light scattering (DLS) and *Z*-potential were measured using a Zetasizer Nanoseries spectrophotometer (Malvern instruments) at 25 °C. TEM samples were prepared on carbon-coated copper grids (200 mesh, Agar scientific) and TEM images were acquired using a Tecnai T20 instrument (FEI). Powder-XRD data were obtained on a Bruker D8 Advance powder diffractometer with a Cu Kα X-ray source (*λ* = 1.54058 Å) operating at 40 kV and 40 mA and a Sol-X detector.

### Synthesis of OA–BaYbF_5_ nanocrystals

OA-capped BaYbF_5_ nanocrystals were prepared according to a published procedure and then dispersed in chloroform.^27^ The resulting NPs were analyzed using TEM, FT-IR spectroscopy, TGA and powder XRD.

### Synthesis of PEG(5)-BP–BaYbF_5_ nanoparticles

PEG(5)-BP was synthesized according to a published procedure.^33^ A displacement reaction was performed by mixing OA–BaYbF_5_ (40 mg) with PEG(5)-BP (80 mg) in chloroform and sonicating the reaction mixture until complete solvent evaporation (≈1 h). The remaining residue was then dispersed in water and washed several times with hexane to remove the displaced oleic acid. Several centrifugations using Vivaspin2 centrifugal filters (MWCO 10 kDa, Vivaproducts) were performed to remove unbound PEG(5)-BP (34.5 mg). The resulting NPs were characterized using TEM, DLS, IR, TGA and *Z*-potential measurements.

### Cell culture and differentiation

The monocytic cell line U937 was grown in suspension in RPMI-1640 cell culture medium supplemented with 10% fetal bovine serum (FBS), 2 mM l-glutamine and 1% antibiotic solution (100 U penicillin/0.1 mg mL^−1^ streptomycin) in a humidified atmosphere of 5% CO_2_ at 37 °C. U937 cells were differentiated into macrophages on 96-well plates by incubating 12 500 cells per well in complete medium supplemented with 4 nM PMA for a total of 4 days. The cell health assay was performed in differentiated macrophages following exposure to PEG(5)-BP–BaYBF_5_ nanoparticles, BaCl_2_, YbCl_3_, and PEG(5)-BP controls and Iohexol in a concentration range of 30–0.01 mM. As a control for cell culture medium dilution with the sample vehicle, a dilution of medium with sterile water in the ratios of 1 : 6 (*ca.* 16% H_2_O) and 1 : 15 (*ca.* 6% H_2_O) was included. After nanoparticles and controls were added, the cells were incubated for 24 hours in a humidified atmosphere of 5% CO_2_ at 37 °C. Then, positive controls for impaired mitochondrial activity (FCCP at 2 mM) and for membrane permeability (TritonX-100 0.1%) were added and cells were incubated for 15 minutes at 37 °C. In sequence, a dye cocktail (100 μL) was added to each well containing Mitotracker Red, Hoechst 33342 and ImageItDead dyes (600 nM, 32 μM and 50 nM final concentrations) and incubated at 37 °C for 30 minutes. Following incubation, cells were carefully washed twice with warm PBS and fixed with PBS containing 4% paraformaldehyde and 5% sucrose. Cells were then treated with CellMask Deep Red dye (2 μg mL^−1^ final concentration), and incubated initially for 120 min at room temperature and then stored at 4 °C until imaging. Immediately prior to imaging, the cells were washed twice in PBS and 100 μL of fresh PBS was added to each well. Plates were imaged using an IN Cell 6000 confocal microscope for high content cell analysis (INCA6000; GE Healthcare) using a 40 ×/60 magnifying objective and acquiring 12 random fields per well. Images were acquired in the UV channel at 405 nm excitation with 455/50 nm and 682/60 emission filters, in the FITC channel at 488 nm excitation with 524/48 nm emission filters, in the dsRed channel at 561 nm excitation with 605/52 nm emission filters and in the Cy5 channel at 642 nm excitation with 682/60 nm emission filters. Automated image analysis was conducted using the IN Cell Developer 1.9 software (GE Healthcare) using custom-developed analysis protocols. Representative images from these studies are shown in the ESI† (Fig. S4).

### CT imaging

Clinical phantom CT images were acquired on a Philips iCT scanner, using the following imaging parameters: thickness, 0.9 mm; pitch, 0.99; 120 kVp, 300 mA; field of view, 350 mm; gantry rotation time, 0.5 s; table speed, 158.9 mm s^−1^. The influence of the operating voltage (80–140 kVp) on the X-ray attenuation of PEG(5)-BP–BaYBF_5_ was investigated. Preclinical CT imaging was performed using a NanoPET-CT™ scanner (Mediso) with a 55 kVp tube voltage and 1100 ms exposure time in 360 projections.

*In vivo* studies were performed in accordance with British Home Office regulations governing animal experimentation and approved by Home Office Project Licence PPL 70/8230. Two female BALB/c mice (10–12 weeks old) were anesthetized with isoflurane and kept under anesthesia for the duration of the experiment (3.5 hours). A pre-contrast CT scan was performed, followed by *i.v.* injection of saline-dispersed PEG(5)-BP–BaYbF_5_ NPs (0.133 M, 100 μL) through the tail vein. Subsequent CT scans (30 min. scans over 3 hours) were then performed, after which the mouse was allowed to recover from anesthesia. The imaging was performed again 24 h post-injection (30 min).

## Abbreviations

BP: Bisphosphonate
CT: Computed tomography
CTA: CT contrast agent for angiography
DLS: Dynamic light scattering
EPR: Enhanced permeation retention
FCCP: Carbonyl cyanide 4-(trifluoromethoxy)phenylhydrazone
FT-IR: Fourier transformed infrared
MPS: Mononuclear phagocytic system
MRI: Magnetic resonance imaging
MWCO: Molecular weight cut off
NP: Nanoparticles
OA: Oleic acid
PAA: Polyacrylic acid
PBS: Phosphate buffered saline
PEG: Polyethylene glycol
PMA: Phorbol 12-myristate 13-acetate
RGD: Arginine–glycine–aspartate
TEM: Transmission electron microscopy
TGA: Termogravimetric analysis
XRD: X-ray diffraction

## Conflicts of interest

There are no conflicts to declare.

## Supplementary Material

TB-008-D0TB00969E-s001
