# Supplementary material for: Synthesis and in vivo evaluation of PEG-BP–BaYbF5 nanoparticles for computed tomography imaging and their toxicity
Source: J Mater Chem B. 2020 Jul 11;8(34):7723–32. doi: 10.1039/d0tb00969e (PMC8693452; doi:10.1039/d0tb00969e)
Supplement: TB-008-D0TB00969E-s001 [file TB-008-D0TB00969E-s001.pdf]

Electronic Supplementary Material (ESI) for Journal of Materials Chemistry B.
This journal is © The Royal Society of Chemistry 2020

Electronic Supplementary Information

Synthesis and *in vivo* Evaluation of PEG-BP-$BaYbF_5$ Nanoparticles for Computed Tomography Imaging and their Toxicity

Cinzia Imberti,[a] Thais Fedatto Abelha,[b] Yong Yan,[c] Jaclyn Lange,[a] Xianjin Cui,[a] Istvan Szanda,[a] Vicky Goh,[d] Lea Ann Dailey,[e] and Rafael T. M. de Rosales [a, *]

[a] Department of Imaging Chemistry & Biology, School of Biomedical Engineering & Imaging Sciences, King's College London, St Thomas' Hospital, London, SE1 7EH, United Kingdom
E-mail: rafael.torres@kcl.ac.uk

[b] School of Pharmacy, University of Nottingham, University Park, Nottingham, NG7 2RD, United Kingdom

[c] School of Chemistry, University of Nottingham, University Park, Nottingham, NG7 2RD, United Kingdom

[d] Department of Cancer Imaging, School of Biomedical Engineering & Imaging Sciences, King's College London, St Thomas' Hospital, SE1 7EH, London, United Kingdom

[e] Department of Pharmaceutical Technology and Biopharmacy, University of Vienna, Althanstraße 14, 1090 Vienna, Austria

Content

S1. High resolution TEM images

S2. TGA for OA-$BaYbF_5$ and PEG(5)-BP-$BaYbF_5$

S3. PEG(5)-BP-$BaYbF_5$ PEG density calculations

S4. Representative fluorescence microscopy images from the *in vitro* toxicity studies.

S1. High resolution TEM images

OA-$BaYbF_5$

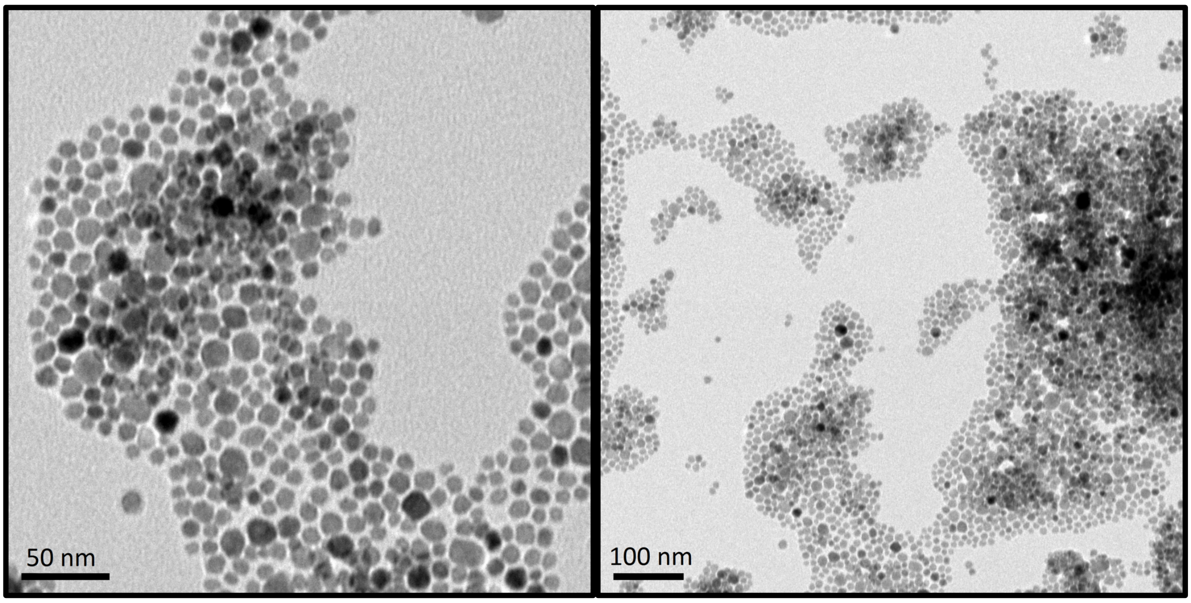

PEG(5)-BP-$BaYbF_5$

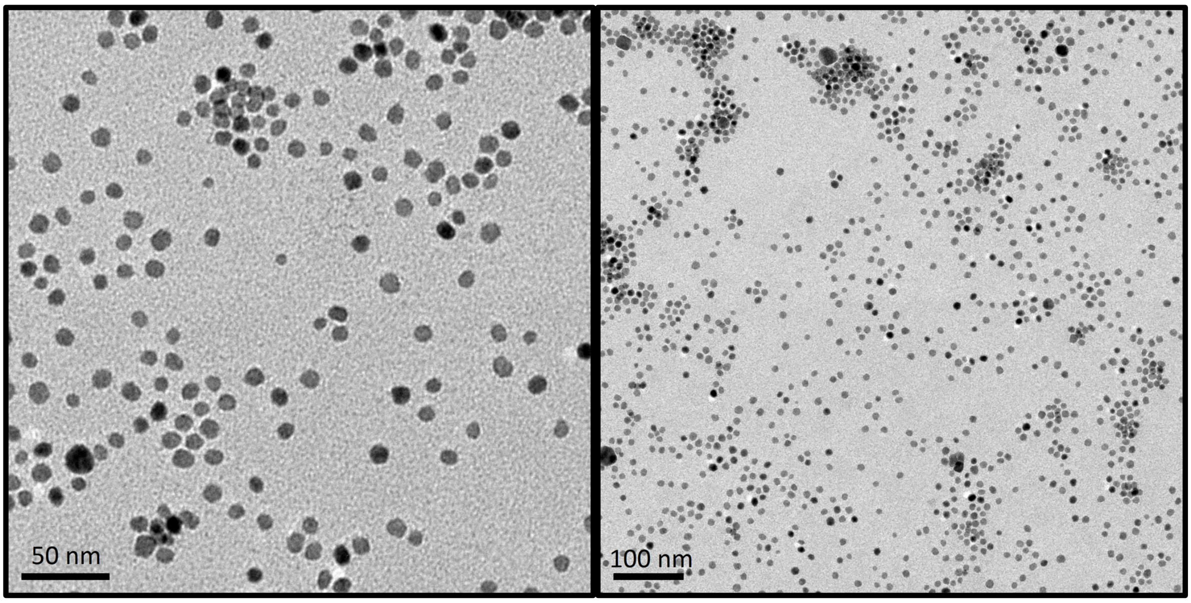

S2. TGA for OA-$BaYbF_5$ and PEG(5)-BP-$BaYbF_5$

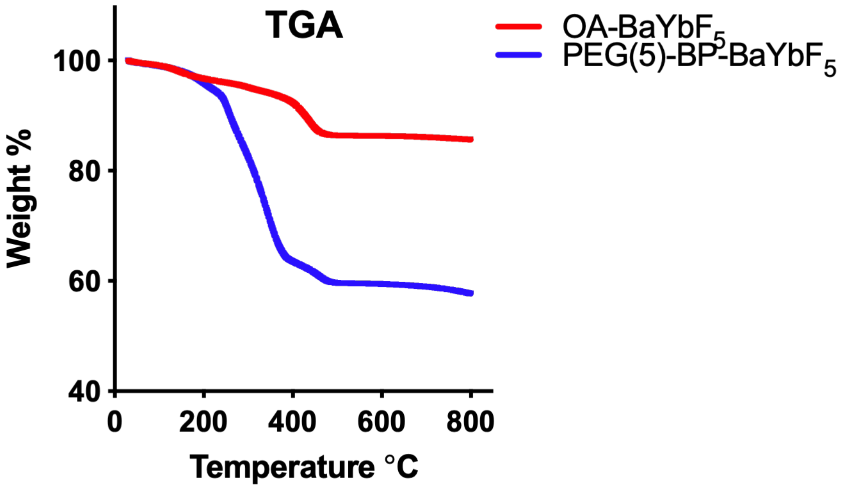

Figure 1. TGA measurements performed for OA-capped and PEGylated $BaYBF_5$ NPs.

S3. PEG(5)-BP-$BaYbF_5$ PEG density calculations

Calculations of the number of PEG(5)-BP molecules bound to each NP were performed assuming the NPs are spheres of a 9.5 nm diameter resulting in a NP volume of 4.49 x 10^{-19} cm^3, and composed of $BaYbF_5$. Density of $BaYBF_5$ was estimated to be the weighted average of YbF_3 and BaF_2 density (6.55 g/cm^3). Based on these assumptions, the mass of a NP $BaYbF_5$ core was estimated to be 2.94 x10^{-18} g.

From TGA measurements

Based on the weight losses measured we can calculate that 37% of PEG(5)-BP-$BaYBF_5$ are due to PEG(5)-BP. This will result in 2.96 mg of PEG(5) and 5.04 mg of $BaYbF_5$ for a 8.00 mg PEG(5)-BP-BaYBf5 TGA sample, corresponding to (2.96x 10^{-3} g/5307 g/mol) x NA = 335.9 x 10^{15} molecules of PEG and (5.04x 10^{-3} g /2.94 x10^{-18} g/NP) =1.71 x 10^{15} Nanoparticles. Hence, the number of PEG molecules per nanoparticle is calculated to be **196**.

Calculation of theoretical maximum density of PEG(5)-BP/NPs

The surface area of a 9.5 nm sphere is 283.4 nm^2. The footprint of a BP is approximately 0.85 nm2 (calculated from a computational model using Chem3D, Cambridge Software). Using these values, the theoretical maximum number of BPs

that can bind to a 9.5 nm sphere is **333**.

Maximum number of PEG(5)-BP/NPs calculated using the amount of non-bound PEG(5)-BP after purification

Based on a scaled up version of the reaction in which 40 mg of OA- $BaYBF_5$ Nanoparticles were reacted with 80 mg of PEG(5)-BP.

- Calculation of number of Yb atoms/NPs

The number of Yb atoms in a nanoparticle is equal to the number of BaYbF5 units. This can be calculated as (NPs mass x Nav / MW $BaYbF_5$ = 2.94 x10^{-18} g x 6.022 x10^{23} atoms/mol /405.32 g/mol) Resulting in 4366.5 Yb atoms/NP

- Calculation of number of $BaYBF_5$ NPs

The Yb concentration of the PEG(5)-BP-$BaYBF_5$ dispersion was measured to be 24.55 mM by ICP-MS, which corresponds to 9.82x 10^{-5} moles and 5.19 x 10^{19} Yb Atoms in 4 mL. Hence, 5.19 x 10^{19} Yb atoms/4366.5 atoms/NP = 1.35 x 10^{16}NPs

- Calculation of number of PEG molecules bound to $BaYBF_5$ NPs

Using the mass of the non-bound PEG(5)-BP, obtained from the washings during purification of the pegylated nanoparticles allowed us to calculate that a maximum of 46.5 mg of PEG(5)-BP was bound to the $BaYBF_5$ NPs.

Hence, using the average MW of PEG(5)-BP value of 5307 g/mol, we can calculate that this corresponds to a maximum of approximately 5.28 x 10^{18} PEG(5)-BP molecules.

- Calculation of number of PEGs/NPs

The maximum number of PEG molecules per NPs is calculated to be 5.28 x 10^{18} / 1.35 x 10^{16} = **389.6** PEG(5)-BP/NP

Since the maximum PEG(5)-BP/NPs density calculated using the recovered PEG(5)-BP was higher than the maximum theoretical density, the latter was deemed more accurate and used for comparison with value obtained from TGA measurements. Based on these value the density of PEG(5)-BP molecules on the surface of the NPs was 58.85% of the maximum theoretical density, which is consistent with the PEG moieties being arranged in a brush regime.

S4. Representative microscopy images from the *in vitro* toxicity studies.

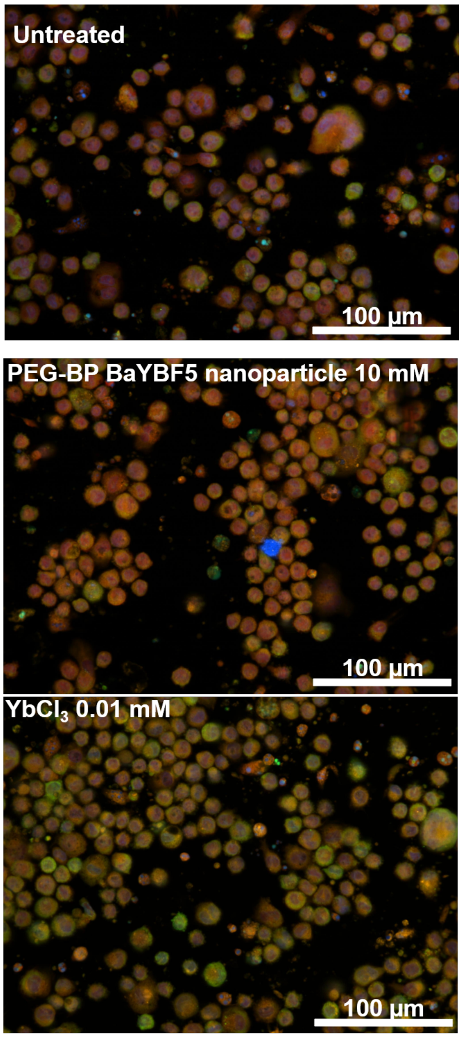

Representative images of **(A)** untreated U937 cells, compared to those treated with **(B)** 10 mM PEG(5)-BP-BaYbF5 nanoparticles and **(C)** 0.01 mM $YbCl_3$. The dye cocktail used contained Mitotracker Red (red) and ImageItDead (green) dyes.
